# Supplementary material for: Exploiting Synthetic Lethality between Germline BRCA1 Haploinsufficiency and PARP Inhibition in JAK2V617F-Positive Myeloproliferative Neoplasms
Source: Int J Mol Sci. 2023 Dec 16;24(24):17560. doi: 10.3390/ijms242417560 (PMC10743753; doi:10.3390/ijms242417560)
Supplement: Supplementary file 1 [file ijms-24-17560-s001.zip › ijms-2697325-supplementary.pdf]

Exploiting synthetic lethality between germline BRCA1 haploinsufficiency and PARP inhibition in JAK2V617F-positive myeloproliferative neoplasms

Max Bermes[1,2], Maria Jimena Rodriguez[1,2], Marcelo Augusto Szymanski de Toledo[1,2], Sabrina Ernst[3], Gerhard Müller-Newen[4], Tim Henrik Brümmendorf[1,2], Nicolas Chatain[1,2], Steffen Koschmieder[1,2,*], Julian Baumeister[1,2,*]

[1]Department of Hematology, Oncology, Hemostaseology, and Stem Cell Transplantation, Faculty of Medicine, RWTH Aachen University, Aachen, Germany

[2]Center for Integrated Oncology Aachen Bonn Cologne Düsseldorf (CIO ABCD), Aachen, Germany

[3]Confocal Microscopy Facility, Interdisciplinary Center for Clinical Research IZKF, RWTH Aachen University, Aachen, Germany

[4]Department of Biochemistry, Faculty of Medicine, RWTH Aachen University, Aachen, Germany

*Contributed equally

Corresponding Author:

Univ.-Prof. Dr. med. Steffen Koschmieder, Department of Hematology, Oncology, Hemostaseology, and Stem Cell Transplantation, Faculty of Medicine, RWTH Aachen University, Pauwelsstr. 30, D-52074 Aachen, Germany, Phone: +49-241-8036102; E-Mail: skoschmieder@ukaachen.de

Running Title:

Synthetic Lethality: BRCA1 & PARP in MPN

Keywords:

Myeloproliferative neoplasms (MPN), BRCA1 haploinsufficiency, heterozygous mutation, olaparib, interferon-alpha, DNA repair, homologous recombination, genomic instability, synthetic lethality.

Abbreviations:

ATM: ataxia telangiectasia mutated, DSB: double-strand break, cGAS-STING: cyclic GMP-AMP synthase-stimulator of interferon genes, HRR: homologous recombination repair, γH2AX: phosphorylated histone 2AX, ISGs: interferon-stimulated genes, MPN: myeloproliferative neoplasms, PARP: poly ADP ribose polymerase, STING: stimulator of interferon genes

Supplements

Supplemental Figures

Figure S1

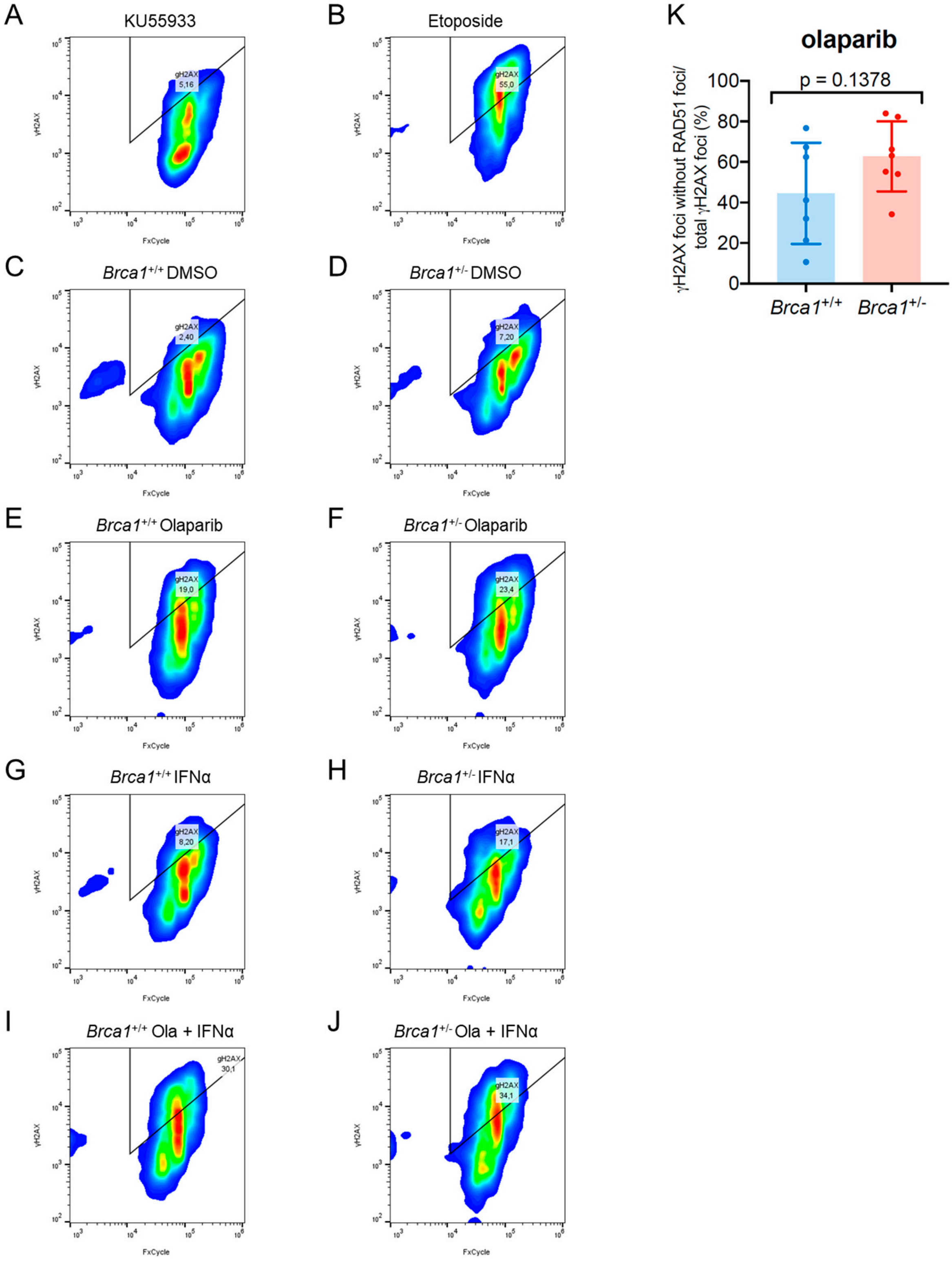

Figure S1: Double-strand break analysis using γH2AX in *Jak2*V617F *Brca1*$^{+/+}$ and *Brca1*$^{+/-}$ cells. **(A-J)** Exemplary data from experiment depicted in Fig. 1A. **(A)** ATM inhibitor Ku55933 as negative control [KU55933; 10 µM], **(B)** topoisomerase II inhibitor etoposide [10 µM] as positive control, **(C+D)** DMSO, **(E+F)** olaparib [10 µM], **(G+H)** IFNα [10 000 U/ml] or **(I+J)** the combination. After treatment, the cells were stained with a phospho-histone H2A.X (Ser139) monoclonal antibody and FxCycle™ Violet stain and then analyzed by flow cytometry (exemplary data from one experiment shown). **(K)** Immunofluorescence analysis of the ratio of γH2AX foci without a corresponding RAD51 focus, relative to the total number of γH2AX foci from data shown in Figure 2A.

Figure S2

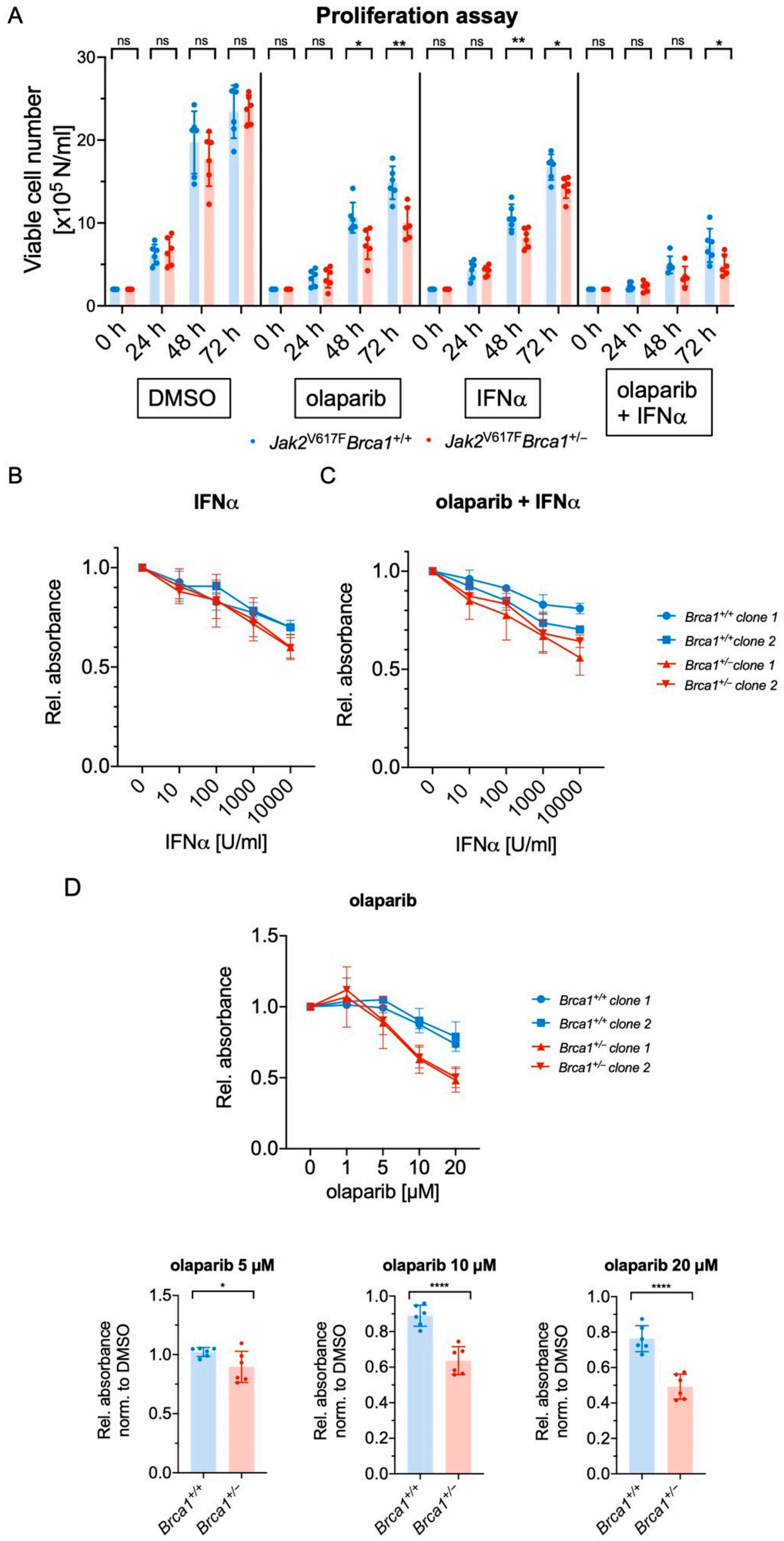
A
Proliferation assay
Viable cell number [x10^5 N/ml]
ns ns ns ns ns ns * ** ns ns ** * ns ns ns *
0 h 24 h 48 h 72 h
DMSO
olaparib
IFNα
olaparib + IFNα
Jak2^V617F Brca1^+/+
Jak2^V617F Brca1^+/–
B
IFNα
Rel. absorbance
IFNα [U/ml]
C
olaparib + IFNα
Rel. absorbance
IFNα [U/ml]
Brca1^+/+ clone 1
Brca1^+/+ clone 2
Brca1^+/– clone 1
Brca1^+/– clone 2
D
olaparib
Rel. absorbance
olaparib [µM]
Brca1^+/+ clone 1
Brca1^+/+ clone 2
Brca1^+/– clone 1
Brca1^+/– clone 2
olaparib 5 µM
olaparib 10 µM
olaparib 20 µM
Rel. absorbance norm. to DMSO
*
****
****
Brca1^+/+
Brca1^+/–

Figure S2: Olaparib and IFNα reduce viable cell numbers and metabolic activity preferentially in *Jak2*V617F *Brca1*$^{+/-}$ cells. **(A)** Cell proliferation was analyzed together with cell viability (see Fig. 3A) in *Jak2*V617F *Brca1*$^{+/+}$ and *Brca1*$^{+/-}$ cells treated with olaparib [10 µM], IFNα [10 000 U/ml] and DMSO by counting the cells after 24 h, 48 h and 72 h with a CASY cell counter (n = 3 with two clones each). **(B+C)** Metabolic activity was assessed by MTT assays in *Jak2*V617F *Brca1*$^{+/+}$ and *Brca1*$^{+/-}$ cells after treatment for 48 h with DMSO or increasing concentrations of IFNα **(B)** alone or **(C)** in combination with an additional fixed concentration of olaparib [10 µM] (B) (n = 3 each). **(D)** Metabolic activity was assessed by MTT assays in *Jak2*V617F *Brca1*$^{+/+}$ and *Brca1*$^{+/-}$ cells after treatment for 48 h with DMSO or increasing concentrations of olaparib (n = 3). Data are presented as mean ± SD and significances defined as: *p<0.05, **p<0.01, ***p<0.001, and ****p<0.0001.

Figure S3

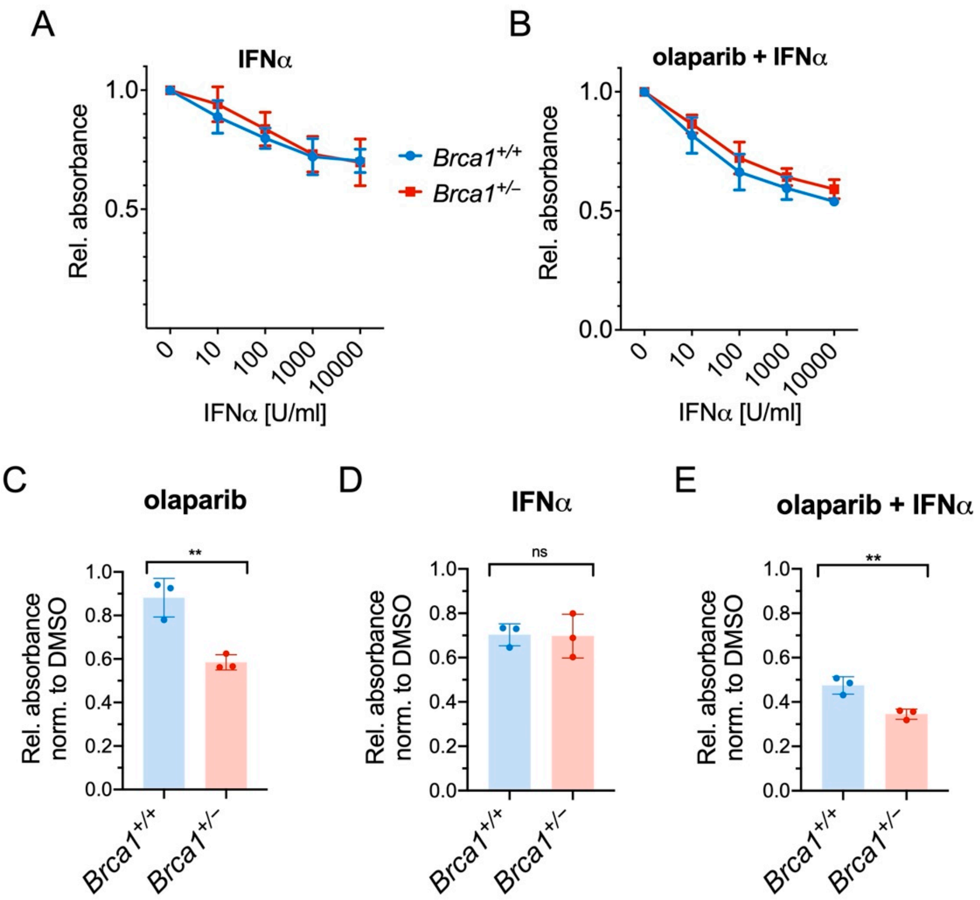

Figure S3: Olaparib but not IFNα has a stronger effect on *Jak2*WT *Brca1*$^{+/-}$ cells than on J*ak2*WT *Brca1*$^{+/+}$ cells. **(A-E)** Metabolic activity was measured by MTT assay in *Jak2*WT *Brca1*$^{+/+}$ and *Brca1*$^{+/-}$ cells after treatment for 48 h with DMSO or increasing concentrations of IFNα (n = 3) without **(A)** or with **(B)** a fixed concentration of olaparib [10 µM]. **(C-E)** Statistical analysis of **(C)** olaparib [10 µM], **(D)** IFNα [10 000 U/ml] and **(E)** the combination of olaparib

[10 µM] and IFNα [10,000 U/ml], normalized to DMSO+H_2O controls. Significances are defined as: *p<0.05 and **p<0.01.

Figure S4

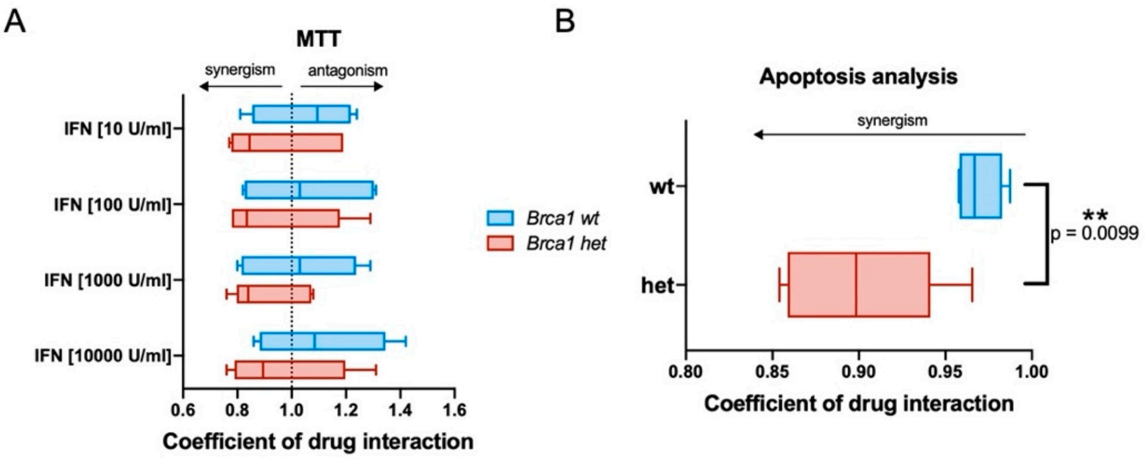

Figure S4: IFNα and olaparib exert stronger synergistic effects on *Brca1*$^{+/-}$ cells than *Brca*1$^{+/+}$ cells. The coefficient of drug interaction (CDI) was calculated for previously described MTT assay with the different concentrations of IFNα combined with olaparib [10 µM] **(A)** and for the apoptosis analysis by flow cytometry with olaparib [10 µM] and IFNα [10,000 U/ml] **(B)**. Significances are defined as: **p<0.01.

Figure S5

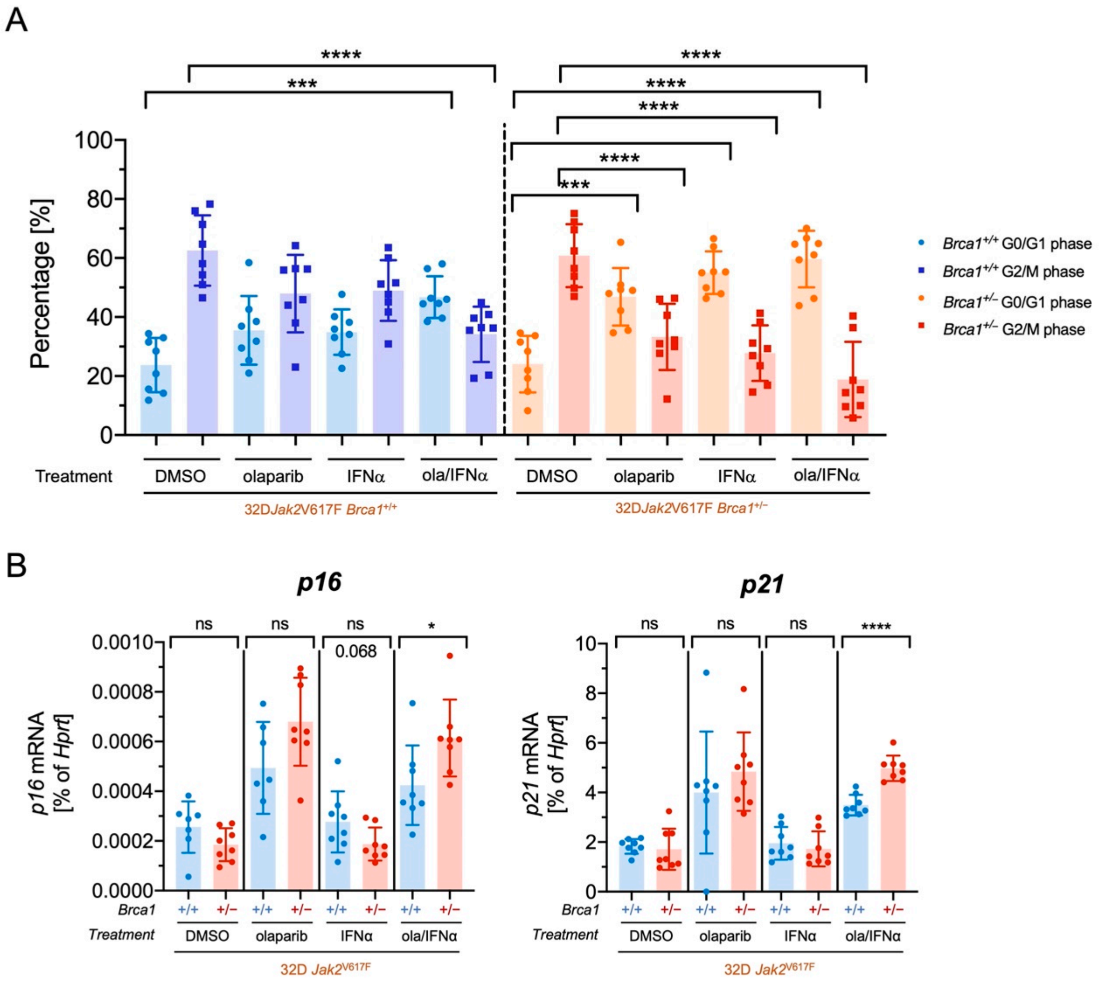

Figure S5: Cell cycle is arrested in G_0/G_1 phase upon treatment with olaparib/IFNα in *Jak2*V617F *Brca1*$^{+/-}$ cells. **(A)** Flow cytometry analysis of G_0/G_1 and G_2/M cell cycle stages using the FxCycle Violet signal from the γH2AX flow cytometry experiments in Fig. 1A in *Jak2*V617F *Brca1*$^{+/+}$ and *Brca1*$^{+/-}$ cells treated with olaparib [10 µM], IFNα [10,000 U/ml], their combination or DMSO for 24 h (n = 4). **(B)** Gene expression of negative cell cycle regulators p16 and p21 was analyzed by RT-qPCR in *Jak2*V617F *Brca1*$^{+/-}$ and cells after 24 h of treatment with olaparib [10 µM], IFNα [10,000 U/ml], the combination of olaparib [10 µM] and IFNα [10,000 U/ml] or DMSO (n = 4). Significances are defined as: *p<0.05, **p<0.01, ***p<0.001 and ****p<0.0001.

Supplemental Tables

Table S1: Comparison of the impact of olaparib and IFNα on metabolic activity in 32D *Jak2*WT and *Jak2*V617F cells with or without a *Brca1* haploinsufficiency. The values are indicated as mean ± SD.

**Relative absorption (%)**	Cell clone			
Treatment	32D *Jak2*wt		32D *Jak2*V617F	
	Brca1 +/+	*Brca1* +/-	*Brca1* +/+	*Brca1* +/-
Olaparib [10 µM]	0.881 ± 0.089	0.584 ± 0.035	0.446 ± 0.059	0.329 ± 0.067
IFNα [10,000 U/ml]	0.703 ± 0.049	0.697 ± 0.098	0.700 ± 0.021	0.601 ± 0.052
Olaparib [10 µM] + IFNα [10,000 U/ml]	0.474 ± 0.039	0.345 ± 0.022	0.335 ± 0.036	0.198 ± 0.053

Materials

Table S2: Antibodies

**Antibody**	**Cat. #/RRID**	**Appl.*/Dilution**	**Company**
Anti-Hu/Mo Phospho-Histone H2A.X (Ser139) Monoclonal Antibody	AB_2574485	FC / 1:100	Invitrogen (Waltham, Massachusetts, USA)
Poly/Mono-ADP Ribose (E6F6A) Rabbit mAb	83732S	WB / 1:1000	Cell Signaling (Danvers, Massachusetts, USA)
GAPDH (6C5) sc-32233, lot # D3018, mouse monoclonal IgG1	sc-32233	WB / 1:1000	Santa Cruz Biotechnology (Dallas, Texas, USA)
P-Histone H2A.X (S139) (20E3) Rabbit mAb	9718T	WB, CM /1:1000, 1:500	Cell Signaling
Polyclonal Goat Anti-Rabbit Immunoglobulin HRP	P0448	WB / 1:2000	Dako (Glostrup, Denmark)
Polyclonal Goat Anti-Mouse Immunoglobulin HRP	P0447	WB / 1:2000	Dako
RAD51 Monoclonal Antibody (14B4)	MA1-23271	CM / 1:500	Invitrogen
Goat anti-Mouse IgG H&L (Alexa Fluor® 488)	ab150113	CM / 1:200	Abcam (Cambridge, England)

Goat anti-Rabbit IgG (H+L) Highly Cross-Adsorbed Secondary Antibody, Alexa Fluor™ Plus 594	A-11012	CM / 1:200	Invitrogen

*Application:
FC = flow cytometry
WB = western blot
CM = confocal microscopy

Table S3: RT-qPCR primer sequences

**Target gene**	**Sequence**
mu Hprt fwd	GGGGGCTATAAGTTCTTTGC
mu Hprt rev	TCCAACACTTCGAGAGGTCC
mu p16 fwd	AAAGCGAACTCGAGGAGAGC
mu p16 rev	TCATCATCACCTGAATCGGGG
mu p21 fwd	GTGGGTCTGACTCCAGCCC
mu p21 rev	CCTTCTCGTGAGACGCTTAC
mu Stat1 fwd	GATCGCTTGCCCAACTCTTG
mu Stat1 rev	ACTGTGACATCCTTGGGCTG
mu Irf7 fwd	TGCTTTCTAGTGATGCCGGG
mu Irf7 rev	CAAGGCTGCGCTCAGGA
mu Sting1 fwd	AGTCCTGCTAGGTGTCCACT
mu Sting1 rev	TGCTTCCTAGACCGGTGTTC
mu Oas1a fwd	ATCAGCCGTCAATGTCGTGT
mu Oas1a rev	CTTGAGTGTGGTGCCTTTGC
mu Isg15 fwd	TGGTACAGAACTGCAGCGAG
mu Isg15 rev	AGCCAGAACTGGTCTTCGTG
mu Mx1 fwd	CCTGGAGGAGCAGAGTGACAC
mu Mx1 rev	GGTTAATCGGAGAATTTGGCAA
hu MT-ATP6 fwd	CGTACGCCTAACCGCTAACA
hu MT-ATP6 rev	AGGCGACAGCGATTTCTAGG
hu STAT1 fwd	TGTATGCCATCCTCGAGAGC
hu STAT1 rev	AGACATCCTGCCACCTTGTG
hu IFIT2 fwd	AGCGAAGGTGTGCTTTGAGA
hu IFIT2 rev	GAGGGTCAATGGCGTTCTGA
hu IFIT3 fwd	TGAAGAACAAATCAGCCTGGTCAC
hu IFIT3 rev	TGACCTCACTCATGACTGCCC

mu Cd52 fwd	AAAGAGTGGGGCCTCATCCA
mu Cd52 rev	TACTGCCGCACACATGACTC
mu Gm34059 fwd	GATGCCCTGATTCCTGTCTAGG
mu Gm34059 rev	GACAACTGCTCTGTCCAAGTC

Methods

Brca1 off-target analysis

DNA was isolated with the Monarch® PCR & DNA Cleanup Kit according to the producer's instructions (New England Biolabs, Ipswich, Massachusetts). Then, for each cell clone, two PCRs were performed with a forward and a reverse primer for CD52 and GM34059. Next, the DNA was purified with the Monarch® PCR & DNA Cleanup Kit and sent for sequencing to Eurofins genomics.

γH2AX flow cytometry

For every clone, $2x10^6$ cells were cultured in 2 ml RPMI medium with 5 ng/ml murine Il-3, 1% Penicillin/Streptomycin and 10% fetal calf serum for 20 h with olaparib [10 µM] or DMSO and IFNα [10 000 U/ml] at 37 °C with. 5% CO_2. Then, either the topoisomerase II inhibitor etoposide [10 µM] was added as positive control for the induction of DSBs or the ATM inhibitor KU55933 [10 µM] was added as negative control and the cells were cultivated for another 4 h. Next, the cells were transferred into a 5 ml polystyrene round bottom tube and centrifuged for 7 min at 400 x g and the supernatant was discarded. Then, 50 µl of Reagent A (Fixation medium) were added and after vortexing, the cells were incubated for 15 min at room temperature. After washing the cells with wash medium (PBS + 2% FCS), centrifugating the samples and discarding the supernatant, 50 µl of Reagent B (Permeabilization medium) and 0.5 µl Phospho-Histone H2A.X (Ser139) Monoclonal Antibody (CR55T33), PE, eBioscience™, (species reactivity: mouse + human) were added. After vortexing, the samples were incubated for 20 min in the dark at room temperature. Again, the samples were washed with wash medium and centrifuged. Then the cells were resuspended in 300 µl wash medium and 0.3 µl of FxCycle™ Violet stain, vortexed and incubated for 30 min in the dark at room temperature. Then, after vortexing the cells, they were analyzed in a Gallios Beckman Coulter flow cytometer. Gating strategy was performed according to Firsanov et al., ("Rapid Detection of γ-H2AX by Flow Cytometry in Cultured Mammalian Cells", *Methods in Molecular Biology (Clifton, N.J.)*, 2017, doi:10.1007/978-1-4939-7187-9_11). This approach is consistent with practices outlined in other relevant publications in the field and taking into account that $FxCycle^{high}$ cells (representing G_2/M phase) exhibit a higher γH2AX signal due to their increased DNA content, even when the relative fraction of DSBs is equivalent to $FxCycle^{int}$ cells (representing G_0/G_1 phase).

**Fluorophore**	**Laser**	**Filter**	**Channel**
GFP	Blue (488 nm)	FL1	525/40
γH2AX-PE	Blue (488 nm)	FL2	575/30
FxCycle™ Violet	Violet (405 nm)	FL9	450/50
